# Distinct phenotype of primary sclerosing cholangitis-associated inflammatory bowel disease

**DOI:** 10.1007/s00535-026-02388-3

**Published:** 2026-03-19

**Authors:** Haruka Okada, Shinya Sugimoto, Atsuto Kayashima, Yohei Mikami, Takanori Kanai, Nobuhiro Nakamoto, Keio PSC–IBD Collaborators, Yusuke Yoshimatsu, Hiroki Kiyohara, Yukie Nakadai, Ryosuke Kasuga, Nobuhito Taniki, Keisuke Ojiro, Shingo Usui, Shogo Sunaga, Ichiro Mizushima, Yuta Kaieda, Shohei Suzuki, Takaya Tabuchi, Makoto Ueno

**Affiliations:** Division of Gastroenterology, Department of Internal Medicine, Keio University School of Medicine, 35 Shinanomachi, Shinjuku-Ku, Tokyo, 160-8582 Japan

**Keywords:** Primary sclerosing cholangitis, Ulcerative colitis, Rectal sparing, Backwash ileitis, Gut–liver axis

## Abstract

Primary sclerosing cholangitis (PSC) is frequently accompanied by colitis with clinicopathologic features that differ from conventional inflammatory bowel disease (IBD). Accumulating evidence indicates that PSC-associated IBD (PSC-IBD) often presents as extensive colitis with a right-sided predominant distribution and characteristic endoscopic features, such as rectal sparing and backwash ileitis. Beyond endoscopic appearance, PSC-IBD also appears to have distinct biological underpinnings, including shared genetic susceptibility, dysregulated bile acid signaling, altered gut microbial communities, and immune crosstalk along the gut–liver axis. These mechanisms may contribute not only to intestinal inflammation but also to PSC-related clinical outcomes. From a long-term perspective, patients with PSC and colitis are consistently classified as a high-risk group for colorectal neoplasia, warranting early and intensive colonoscopic surveillance. In parallel, surveillance for hepatobiliary malignancies remains central in PSC care, although risk stratification continues to evolve. Therapeutic management generally follows established IBD algorithms, yet the extent to which colitis-directed therapies modify PSC outcomes remains uncertain, with heterogeneous findings across cohorts and endpoints. In this review, we summarize current knowledge on the clinical phenotype, mechanistic framework, and outcome-driven management of PSC-IBD, and highlight future directions toward precision surveillance and mechanism-based interventions targeting bile acid–microbiota–immune interactions.

## Introduction

Primary sclerosing cholangitis (PSC) is a rare, chronic cholestatic liver disease characterized by progressive inflammatory and fibrotic changes of the intrahepatic and extrahepatic bile ducts. Despite its relatively indolent course in some patients, PSC often progresses to biliary cirrhosis and liver failure, and liver transplantation (LT) remains the only curative treatment. As a result, PSC continues to represent a disease with substantial unmet clinical needs.

A distinctive feature of PSC is its strong association with inflammatory bowel disease (IBD). Approximately 60–70% of patients with PSC in Western countries have concomitant IBD, most commonly ulcerative colitis (UC). Accumulating evidence indicates that PSC-associated IBD (PSC-IBD) differs from classical UC in terms of clinical presentation, endoscopic features, and long-term outcomes, including an increased risk of colorectal neoplasia. These differences have important implications for disease surveillance and management in patients with PSC. In this review, we summarize current evidence regarding the unique characteristics of PSC-IBD, focusing on its epidemiology, clinical features, long-term outcomes, and management considerations. We also discuss emerging data supporting the concept that PSC-IBD represents a distinct disease phenotype, and consider how this concept should inform future clinical practice.

## Positioning of PSC and PSC-associated IBD

### Overview of PSC

PSC is an idiopathic chronic inflammatory cholangiopathy characterized by multifocal, segmental strictures of the intrahepatic and/or extrahepatic bile ducts. PSC is a rare disease, with reported prevalence ranging from fewer than 1 to 32 per 100,000 population. The prevalence is highest in Northern Europe and North America and gradually decreases toward southern regions with generally low prevalence reported in Asia [1–3]. In Japan, the prevalence of PSC was reported to be 1.80 per 100,000 population in 2018 [4], which represents an increase compared with 0.95 per 100,000 population reported in a 2007 survey [5].

Although PSC is relatively uncommon as an extraintestinal manifestation among patients with IBD—affecting approximately 1.92–3.08% of patients with UC and 0.69–1.28% of those with Crohn’s disease (CD) [6]—IBD is frequently observed in patients with PSC. In Western countries, 60–70% of patients with PSC have concomitant IBD. In contrast, studies from Japan have reported a lower prevalence of IBD among patients with PSC, at approximately 40%, as well as a bimodal age distribution at disease onset in men, suggesting epidemiologic features distinct from those observed in Western populations [7]. Although IBD is often diagnosed prior to PSC, it may also be identified after the onset of PSC or following LT [8, 9]. Magnetic resonance cholangiography (MRCP) is the current gold standard for the diagnosis of PSC. Typical cholangiographic findings include irregularity and multifocal strictures of the intrahepatic and extrahepatic bile ducts, as well as a characteristic beaded appearance in advanced disease [10]. Histologically, PSC is characterized by periductal fibrosis and inflammatory cell infiltration; in classic cases, concentric periductal fibrosis, often termed onion-skin fibrosis, can be identified, although such a classic appearance is relatively uncommon [11].

The clinical course of PSC is highly variable. While some patients remain stable for extended periods, a substantial proportion progress to end-stage cirrhosis and ultimately require LT. The median interval from diagnosis to PSC-related death or LT has been reported to be 10–21 years [12, 13]. At present, LT remains the only curative treatment for PSC. In Japan, where deceased donor availability is limited, PSC continues to pose a significant clinical challenge.

### Epidemiologic association between PSC and colitis

One proposed explanation for the apparently lower prevalence of IBD among patients with PSC in Japan is under-recognition. Colitis associated with PSC often causes mild or no gastrointestinal symptoms, and colonoscopy may therefore be underutilized in asymptomatic individuals. Supporting this possibility, an earlier Japanese study reported that the prevalence of IBD increased from 37 to 61% when analyses were restricted to patients who underwent total colonoscopy [14]. Similar concerns regarding underdiagnosis of concomitant IBD have been raised internationally [15]. Reflecting these concerns, recent Western guidelines recommend routine ileocolonoscopy at the time of PSC diagnosis regardless of gastrointestinal symptoms [11, 16]. The 2024 Japanese guideline similarly emphasizes the importance of evaluating comorbid IBD and recommends proactive ileocolonoscopy irrespective of symptoms [10]. Moreover, recent studies have reported that molecular evidence of intestinal inflammation is detectable in the majority of PSC patients who were previously considered endoscopically normal [17].

PSC-IBD exhibits clinical and endoscopic features distinct from those of classical UC and CD [18]. Multiple cohort studies have also shown that PSC-IBD is associated with an increased risk of colorectal neoplasia, including dysplasia and cancer, compared with both the general population and patients with UC alone [19, 20]. Accordingly, the presence of PSC-IBD represents a critically important factor when considering long-term outcomes and surveillance strategies in patients with PSC.

In recent years, PSC-IBD has been suggested to differ from classical UC not only in endoscopic phenotype but also in genetic background, gut microbiome composition, and bile acid (BA) profiles [2]. These findings raise the possibility that PSC-IBD represents a distinct disease phenotype rather than a simple variant of UC. This conceptual framework provides the basis for subsequent discussions of PSC-IBD pathogenesis, prognosis, and management.

## Clinical and endoscopic phenotype

### Phenotypic features of PSC-IBD

A characteristic endoscopic pattern distinct from conventional UC has been reported in PSC-IBD. This phenotype is typically characterized by extensive colitis accompanied by a proximal-to-distal gradient of inflammation, in which inflammatory activity is greatest in the cecum and ascending colon and becomes attenuated toward the distal colon. This distribution is commonly referred to as right-sided predominance. Two additional hallmark features of PSC-IBD are rectal sparing (RS) and backwash ileitis (BI) [6]. BI refers to UC-like diffuse inflammation confined to a short segment of the terminal ileum that is contiguous with cecal inflammation in extensive colitis. Importantly, BI is not usually associated with features characteristic of CD, such as patchy involvement, deep ulcers, fistulas, or strictures [21, 22]. These findings contrast with the typical patterns of conventional UC, in which inflammation extends continuously from the rectum, and are thought to define a characteristic endoscopic phenotype of PSC-IBD. Although both RS and BI were originally described as atypical inflammatory distributions in UC independent of PSC, their morphologic definitions have been refined in recent pathological studies [23, 24] and incorporated into clinical guidelines [22, 25].

A seminal cohort study by Loftus et al. [18] established PSC-IBD as a distinct clinical phenotype by proposing explicit endoscopic and histologic definitions. In that study, RS was defined as a rectal mucosa that is endoscopically or histologically normal, or less inflamed than the proximal colon, and BI was defined as terminal ileitis accompanied by a patulous ileocecal valve and a granular terminal ileal mucosa. The investigators compared 71 patients with PSC-IBD with 142 age- and sex-matched patients with UC alone, demonstrating that more than 80% of PSC-IBD cases had extensive colitis. Moreover, RS and BI were observed far more frequently in PSC-IBD, than in UC alone (52% vs. 6% and 51% vs. 7%, respectively). Based on these findings, PSC-IBD was proposed as an independent IBD phenotype characterized by extensive colitis with frequent RS and BI. Histopathological studies have further supported the concept that PSC-IBD differs, at least in part, from conventional UC. Comparative analyses of biopsy specimens obtained from the right and left colon have consistently demonstrated a higher frequency and greater activity of inflammation in the right colon [26–28], consistent with endoscopic observation of right-sided predominance. In a recent pediatric PSC-IBD cohort, pathological evaluation revealed a high prevalence of features not incorporated into conventional histologic activity indices for UC, including lamina propria-predominant neutrophilic infiltration and superficial villiform change, particularly in the right colon [29].

Subsequently, multiple cohort studies from Western [8] and Asia [30, 31] have reported higher frequencies of RS and BI in PSC-IBD compared with conventional UC. However, not all studies have demonstrated substantial differences between PSC-IBD and UC alone [8], resulting in heterogeneous findings across cohorts, likely reflecting heterogeneity in study populations, diagnostic criteria, and endoscopic assessment strategies. In a systematic review integrating available evidence, the reported prevalence of RS in PSC-IBD ranged from 6 to 66%, and that of BI ranged from 5 to 46%, underscoring considerable interstudy variability [32].

### Phenotype of PSC-IBD in our single-center cohort

Across previously published cohorts, substantial variability in diagnostic criteria has been noted, particularly in multi-center studies, and the timing of endoscopic assessment is often not clearly specified. To address these limitations, we leveraged a single-center retrospective observational cohort in Japan, conducted at a high-volume tertiary referral center for both PSC and IBD. Importantly, we performed a longitudinal review of all available colonoscopic examinations for each patient, rather than a single cross-sectional assessment, to derive more accurate prevalence estimates and to better characterize the endoscopic phenotype of PSC-IBD.

**Fig. 1:**
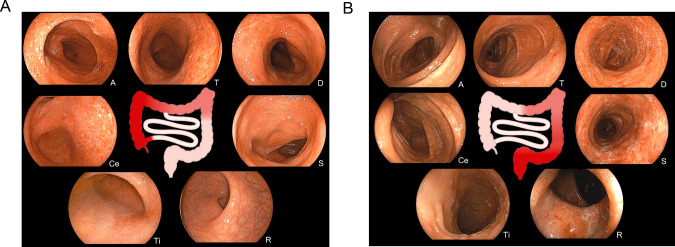
Typical endoscopic findings of primary sclerosing cholangitis-associated inflammatory bowel disease and conventional ulcerative colitis (**A**) Representative endoscopic images of primary sclerosing cholangitis-associated inflammatory bowel disease (**B**) Representative endoscopic images of conventional ulcerative colitis Ti, terminal ileum; Ce, cecum; A, ascending colon; T, transverse colon; D, descending colon; S, sigmoid colon; R, rectum

Among patients with PSC, we identified 126 patients with PSC-IBD and compared them with 126 age- and sex-matched patients selected from a conventional UC cohort of 3,419 patients without PSC (Table 1). Regarding disease extent, the distributions differed markedly between the groups; PSC-IBD was predominantly extensive (97.6%), with few left-sided cases (2.4%) and no proctitis, whereas UC demonstrated a more limited extent (extensive, 66.7%; left-sided, 20.6%; proctitis, 12.7%, *P* < 0.001). RS was observed in 92.1% of patients with PSC-IBD, compared with only 11.1% of those with UC (*P* < 0.001). Similarly, BI was significantly more frequent in patients with PSC-IBD than in those with UC without PSC (20.6% vs. 3.2%, *P* < 0.001). These findings indicate that right-sided predominance, RS, and BI are consistently observed as hallmark endoscopic features of PSC-IBD in an Asian single-center cohort. Notably, our longitudinal evaluation demonstrated substantially higher frequencies of RS and BI than those reported in many previous studies, suggesting that these findings are not sporadic or incidental but rather represent defining characteristics of colitis in patients with PSC. The discrepancy with earlier studies is likely attributable to endoscopic assessments that rely on images obtained predominantly during remission, which may underestimate the true right-sided inflammatory activity in PSC-IBD (Fig. 1).

**Table 1:** Clinical characteristics, endoscopic findings and outcomes of PSC-associated IBD compared to those with UC without PSC

			PSC-IBD^a^	UC^b^	P-value
			(n=126)	(n=126)	
Age, years, median (IQR)^c^			35 (25–45)	35 (25–46)	-
Follow-up duration from PSC diagnosis, years, median (IQR)			6 (2–12)	-	
Male			90 (71.4%)	90 (71.4%)	-
Age at colitis diagnosis, years, median (IQR)			24 (19–33)	20 (16–28)	<0.001
Order of diagnosis					
	PSC→Colitis		18 (14.3%)		
	Colitis→PSC		32 (25.4%)		
	Simultaneous^d^		63 (50.0%)		
	Unknown		13 (10.3%)		
Disease extent^e^					<0.001
	Proctitis (E1)		0 (0%)	16 (12.7%)	
	Left-sided (E2)		3 (2.4%)	26 (20.6%)	
	Extensive (E3)		123 (97.6%)	84 (66.7%)	
Rectal sparing^f^					<0.001
	Yes		116 (92.1%)	14 (11.1%)	
	No		10 (7.9%)	112 (88.9%)	
Backwash ileitis^f^					<0.001
	Yes		26 (20.6%)	4 (3.2%)	
	No		82 (65.1%)	120 (95.2%)	
	Not assessable		18 (14.3%)	2 (1.6%)	
Treatment history^g^					
	Oral 5-aminosalicylic acid		111 (88.1%)	121 (96.0%)	0.034
	Systemic corticosteroid				
		Due to colitis	41 (32.5%)	70 (55.6%)	<0.001
		Due to liver disease	14 (11.1%)	-	
	Cytapheresis		9 (7.1%)	17 (13.5%)	0.150
	Calcineurin inhibitor				
		Due to colitis	7 (5.6%)	15 (11.9%)	0.120
		Due to liver transplantation	7 (5.6%)	-	
	Thiopurine				
		Due to colitis	29 (23.0%)	47 (37.3%)	0.019
		Due to liver disease	7 (5.6%)	-	
	Anti-TNF		20 (15.9%)	35 (27.8%)	0.032
		Infliximab	13	25	
		Adalimumab	10	17	
		Golimumab	2	6	
	Anti-integrin α4β7	Vedolizumab	19 (15.1%)	17 (13.5%)	0.860
	JAK inhibitors		6 (4.8%)	14 (11.1%)	0.100
		Tofacitinib	2	6	
		Upadacitinib	3	7	
		Filgotinib	2	4	
	Anti-IL-12/23 / Anti-IL-23	8 (6.3%)	10 (7.9%)	0.810	
		Ustekinumab	7	8	
		Mirikizumab	2	0	
		Risankizumab	1	2	
	α4 integrin inhibitors	Carotegrast methyl	4 (3.2%)	0 (0%)	0.120
Colitis-associated neoplasia			7 (5.6%)	1 (0.8%)	0.066
Interval from colitis diagnosis to colitis-associated neoplasia, years, median (IQR)			5 (3–12)	23	
Colectomy			4 (3.2%)	5 (4.0%)	>0.999
	Due to neoplasia		3	1	
	Due to disease activity	1	4		
Cholangiocarcinoma		10 (7.9%)	0		
Interval from PSC diagnosis to cholangiocarcinoma, years, median (IQR)		10 (3–13)	-		
Death			13 (10.3%)	0 (0%)	<0.001
	Liver failure		8	0	
	Liver failure after liver transplantation	1	0		
	Cholangiocarcinoma		3	0	
	Colorectal cancer	1	0		

PSC, primary sclerosing cholangitis; IBD, inflammatory bowel disease; PSC-IBD, PSC-associated IBD; UC, ulcerative colitis; IQR, interquartile range; TNF, tumor necrosis factor; JAK, Janus kinase; IL, interleukin

a. Among patients with PSC followed at Keio University Hospital between January 2012 and August 2025, 126 patients with PSC-associated IBD (PSC-IBD) with evaluable ileocolonoscopy were included. Cases in which all available endoscopic images showed complete remission and were therefore insufficient to assess colitis were excluded from the matched case–control analysis. In patients with at least one endoscopic examination demonstrating active disease, all colonoscopy images in the electronic medical record during follow-up, were retrospectively reviewed by at least two endoscopy specialists, including those with expertise in IBD, regardless of whether the examinations were performed during active disease or remission.

b. The comparator group comprised 126 age- and sex-matched patients selected from a conventional UC cohort (n = 3,419) without PSC.

c. For patients who remained under follow-up, age was defined as of January 2025, whereas for those who had discontinued follow-up or had died, age at the last follow-up visit was used.

d. If the second diagnosis was made within one year during the evaluation of the other disease, the two conditions was classified as having a simultaneous diagnosis.

e. The extent of colitis was classified according to the Montreal Classification. The extent of UC was defined as the most proximal involvement of inflammation by endoscopy and/or histology at any time during the study period.

f. Rectal sparing and backwash ileitis were defined as described [18]. Colonoscopy images from patients who had received rectal enema therapy within 6 months prior to the procedure were not classified as rectal sparing. When images of the terminal ileum or ileocecal valve were not available, the finding was recorded as “not assessable.” Based on colonoscopy images available in the medical records, a finding was considered present if rectal sparing or backwash ileitis was observed at least once.

g. Lifetime use of medications

Regarding the temporal relationship between PSC and IBD, simultaneous diagnosis was observed in 50.0% of patients, IBD preceded PSC in 25.4%, and PSC preceded IBD in 14.3%, with median lead-times of 5.5 years (IQR 2–8) and 5 years (IQR 2.5–10.3), respectively. With respect to lifetime medication exposure, 5-aminosalicylic acid (5-ASA), systemic corticosteroids, thiopurines, and tumor necrosis factor (TNF) inhibitors were used more frequently in UC without PSC, consistent with prior reports suggesting that PSC-IBD tends to exhibit lower clinical activity compared with conventional UC [33]. Despite this, all-cause mortality during follow-up was significantly higher in the PSC–IBD group (*P* < 0.001). Taken together, our findings support the established concept that colitis in patients with PSC represents an entity distinct from conventional UC.

## Pathogenesis: PSC as a multifactorial disease and the gut–liver axis

**Fig. 2:**
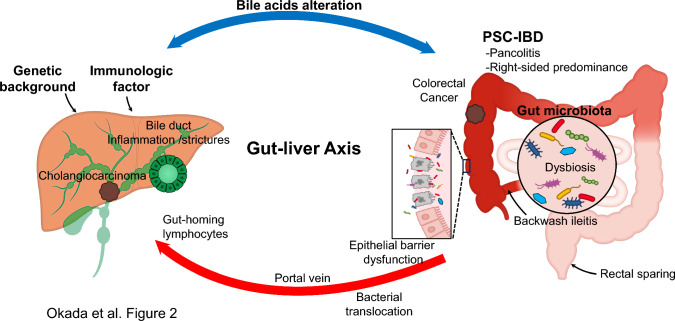
Multifactorial pathogenesis of primary sclerosing cholangitis centered on the gut–liver axis PSC-IBD, primary sclerosing cholangitis-associated inflammatory bowel disease

PSC is widely regarded as a multifactorial disease that cannot be explained by a single pathogenic mechanism. Genetic susceptibility, immune dysregulation, disrupted BA signaling, and altered gut microbiota are thought to interact in a complex manner to drive disease development and progression (Fig. 2). In patients with PSC-IBD, inflammatory networks mediated through the gut–liver axis may contribute not only to biliary injury but also to the characteristic colitis phenotype.

### Genetic background

Evidence for a genetic contribution to PSC susceptibility is supported by studies of familial aggregation, which have demonstrated that first-degree relatives of patients with PSC have a more than tenfold increased risk of developing the disease compared with the general population [34]. Although specific human leukocyte antigen (HLA) alleles, such as HLA-B08 and DRB1*03, have been associated with PSC, a substantial proportion of patients do not carry typical risk alleles [35]. Genome-wide association studies have identified multiple susceptibility loci outside the HLA region [36–38]. These loci include genes involved in innate and adaptive immune responses, BA signaling, and intestinal epithelial barrier function, suggesting the potential involvement of multiple biological networks in PSC pathogenesis.

Shared susceptibility genes have been reported between PSC and IBD [37, 39]. However, the extent of this overlap appears limited; compared with the strong genetic correlation observed between UC and CD, PSC exhibits significantly lower genetic correlation with either condition [38]. These observations suggest that PSC-IBD may also represent a genetically distinct disease phenotype relative to conventional forms of IBD [2, 37].

### Immunologic factors

In PSC, immune activation is supported by the frequent detection of autoantibodies, including perinuclear anti-neutrophil cytoplasmic antibody (p-ANCA) [40], together with portal tract–predominant T cell infiltration in liver tissue [41]. T cell receptor repertoire sequencing has revealed distinct PSC-associated repertoires across chronic liver diseases [42]. Furthermore, paired gut–liver analyses in PSC-IBD have identified memory T cells of common clonal origin [43], supporting involvement of the gut–liver axis. Oligoclonal expansion of bile duct–associated T cell receptor clonotypes further implies chronic antigenic stimulation within the hepatobiliary compartment [44]. Single-cell analyses further indicate expansion of tissue-resident naïve-like CD4 + T cells in PSC livers, which may acquire Th17-related effector functions [45]. Although the relevant target antigens have long remained unclear, recent data suggest involvement of Epstein–Barr virus (EBV) epitope-specific T cell responses restricted by PSC-associated HLA alleles [46].

As a potential mechanism linking intestinal immune activation to hepatobiliary inflammation, molecular determinants governing the selective homing of gut-primed lymphocytes to the liver have received considerable attention. In PSC, ectopic hepatic expression of C–C motif chemokine ligand 25 (CCL25) and mucosal vascular addressin cell adhesion molecule 1 (MAdCAM-1), which are normally selectively expressed in gut-associated lymphoid tissues, has been proposed to enable constitutive recruitment of gut-tropic T cells to the liver via C–C motif chemokine receptor 9 (CCR9) and α4β7 integrin [47, 48]. In addition, recent studies have reported high-frequency detection of autoantibodies against integrin αvβ6 in patients with UC and PSC [49–51]. Further advances in this area are anticipated.

Despite the distinct clinical phenotype of PSC-IBD, robust differences in systemic immune phenotypes compared with conventional IBD have not yet been firmly established. Modest differences have been reported in specific lymphocyte subsets, including interferon-γ–producing T cells [52] and innate lymphoid cells [53]. Notably, in PSC-IBD, accumulation of IL-17A⁺FOXP3⁺ double-positive T cells and clonal IgG plasma cells has been observed in the right colon, suggesting an antigen-driven adaptive immune response. This immune signature appears to differ from that seen in IBD alone and may contribute to the PSC-specific pattern of intestinal inflammation [54]. Collectively, these findings are consistent with a model in which gut microbes may be associated with enhanced antigen presentation, adaptive immune activation, and subsequent inflammation, potentially relating to the right-sided predominant colitis in PSC.

### Bile acid alterations

BAs are synthesized in the liver and secreted into the intestine via the biliary tract. Beyond their role in lipid absorption, BAs act as signaling molecules that regulate metabolic and immune responses through receptors, such as the farnesoid X receptor (FXR) and the transmembrane G protein-coupled receptor 5 (TGR5). In the liver, primary BAs, including cholic acid and chenodeoxycholic acid, are synthesized from cholesterol, conjugated with taurine or glycine, and excreted into bile. In the intestine, a subset of primary BAs is metabolized by gut microbiota into secondary BAs, including deoxycholic acid and lithocholic acid. Most primary and secondary BAs are reabsorbed in the small and large intestines and returned to the liver via the portal circulation as part of the enterohepatic circulation [55, 56]. BA homeostasis is disrupted in PSC as a consequence of cholestasis, with an increased proportion of primary BAs and alterations in specific BA species [57, 58]. Moreover, BA signaling appears to be dysregulated, as evidenced by downregulation of TGR5 in biliary epithelial cells [59], as well as reported alterations in the FXR–fibroblast growth factor 19** (**FGF19) axis [60]. In PSC-IBD, decreased expression of FXR has been observed particularly in the right colon compared with IBD alone or healthy controls [61]. This finding suggests that region-specific disruption of BA signaling may contribute to right-sided inflammation and epithelial barrier dysfunction.

Recent multicenter cohort studies have demonstrated that PSC-IBD differs systematically from IBD alone in its serum BA profile, characterized by reduced sulfated BAs, increased conjugated secondary BAs, and enrichment of bacterial species harboring genes related to BA metabolism [62]. Multiomics analyses of colonic mucosa further revealed selective activation of BA signaling pathways in PSC-associated UC, consistent with microbial functional profiles associated with abnormal BA metabolism [63]. Collectively, these findings support the concept that PSC-IBD represents a distinct inflammatory phenotype from conventional UC**,** potentially associated with dysregulated interactions between BAs and the gut microbiota.

### Gut microbiota

The human gastrointestinal tract is estimated to harbor more than 10 trillion microorganisms spanning approximately 1,000 microbial species [64, 65], and the collective gene repertoire of the gut microbiome is estimated to be approximately 150-fold larger than that of the human genome [66]. These microbes utilize dietary and host-derived nutrients and form a dynamic ecosystem shaped by interspecies interactions and bidirectional host–microbe crosstalk. Because the liver receives more than 70% of its blood supply from the portal vein, it is continuously exposed to gut-derived antigens and microbial products, establishing a close functional link between the intestinal microbiota and hepatic immunity.

Given the high frequency of IBD in PSC, microbial dysbiosis has long been implicated in its pathogenesis, supported by reports that antibiotic therapy can transiently improve clinical and biochemical markers [67]. PSC is characterized by reduced microbial diversity and enrichment of specific genera including *Enterococcus*, *Streptococcus*, and *Veillonella* [68, 69]. Altered bile microbiota [57] and fungal dysbiosis [70] have also been reported. Fecal microbiota analyses have suggested that PSC exhibits a shared dysbiosis irrespective of concomitant IBD, with only a limited impact of associated colitis on the overall microbial community structure [68–70]. In contrast, studies directly comparing the gut microbiota of PSC-IBD with that of conventional UC have identified a distinct microbial profile characterized by reduced diversity together with enrichment of specific genera, raising the possibility that PSC-IBD represents a microbiome phenotype distinct from typical UC [68, 69, 71]. Notably, compositional differences between PSC and UC have been observed not only in fecal microbiota but also in studies of mucosa-associated microbiota, which are considered more closely linked to disease-relevant host–microbe interactions [63, 72]. Moreover, gut microbes may influence PSC not only through the organisms themselves but also through the production of microbial products and metabolites. Indeed, microbial metabolites, such as pyridoxal-5′-phosphate, a vitamin B_6_ derivative, and branched-chain amino acids, have been associated with PSC progression [73].

Whether these microbial alterations are causal or secondary to inflammation remains debated. However, gut microbes have been proposed as a potential source of antigens that mimic self-epitopes, thereby promoting autoimmune responses [74, 75]. Supporting a gut–liver axis mechanism, T cells activated by microbial antigens in the intestine migrate to the liver and are implicated in immune injury [47, 76]. Approximately 15% of liver-infiltrating T cells have been shown to share identical T cell receptors with intestinal T cells and to retain responsiveness to the same antigen [43]. Moreover, our group demonstrated that fecal microbiota transplantation from patients with PSC into germ-free mice can induce PSC-like sclerosing cholangitis via hepatic Th17 responses, suggesting that specific microbial communities can directly contribute to disease initiation in this model [77]. Notably, *Klebsiella pneumoniae* and *Enterococcus gallinarum* with bacterial translocation capacity were detected in mesenteric lymph nodes of mice transplanted with stool samples from a patient of PSC, and were also frequently identified in stool samples from patients with PSC [78]. Carriage of both organisms was associated with higher disease activity and poorer transplant-free survival in Japanese [78] and Norwegian cohorts [79], highlighting their potential clinical utility as diagnostic biomarkers and therapeutic targets in PSC. In the same cohort, detection of *Klebsiella* in the ascending colon was associated with significantly earlier recurrence after LT, underscoring the importance of intraluminal localization of specific taxa.

Despite rapid methodological advances in microbiome research, substantial gaps remain in our understanding of the mechanisms linking dysbiosis to the disease initiation and progression, and further studies are warranted.

## Long-term outcomes and management

### Impact of concomitant colitis on the clinical course of PSC

Evidence regarding the relationship between colitis activity in PSC-IBD and the severity or clinical course of PSC is mixed. While many patients with PSC-IBD remain in clinical remission or exhibit only mild colitis over prolonged periods, biliary lesions may nonetheless progress [80]. In addition, among patients with advanced PSC requiring LT, UC has been reported to relapse less frequently and to require lower use of corticosteroids, thiopurines, or surgical intervention, accompanied by milder histologic inflammation [81]. These observations suggest that the severity of colitis and the progression of biliary disease do not necessarily parallel each other.

Conversely, in patients with PSC after LT, the risk of recurrent PSC (rPSC) has been reported to be markedly higher in those who experienced moderate-to-severe IBD activity [82]. Another study showed that, among patients with PSC-IBD and elevated fecal calprotectin levels, the risk of biliary events—such as cholangitis or the need for endoscopic retrograde cholangiopancreatography (ERCP) interventions—was substantially higher than the risk of colitis flares [83]. These findings indicate that intestinal inflammatory burden may still influence biliary disease in at least a subset of patients.

If PSC-IBD were merely an intestinal manifestation of PSC as a systemic disease, colitis activity would be expected to parallel PSC activity more consistently, and colitis-directed interventions would be expected to exert uniform effects on PSC progression. However, available clinical evidence has not consistently demonstrated such patterns, and reported associations between intestinal inflammatory burden, colitis-directed interventions, and PSC outcomes remain heterogeneous.

### Association between surgery and prognosis in PSC

Evidence regarding the impact of colectomy on PSC risk and long-term outcomes remains mixed, and variation in surgical indications and techniques across eras and centers complicates causal inference. In a Swedish UC cohort, comparison of patients who underwent colectomy showed no difference in subsequent PSC risk, leading the authors to conclude that colectomy does not prevent PSC development [84]. In contrast, another cohort study reported that patients who underwent colectomy before PSC diagnosis had longer transplant-free survival than those without prior colectomy at diagnosis, whereas colectomy performed after PSC diagnosis was not associated with a clear prognostic benefit [85]. These findings raise the possibility that colitis may contribute to PSC particularly in an early, promotive phase of disease development [86]. After restorative surgery with ileal pouch–anal anastomosis, ongoing intestinal inflammation may persist despite colectomy. In this context, pouchitis occurs substantially more frequently in UC with PSC than in UC alone [87]. Patients with PSC have also been reported to be more likely to develop chronic pouchitis and to exhibit reduced responsiveness to antibiotic therapy [88].

Because more than one quarter of patients develop rPSC within 10 years after LT, the post-transplant course is often used as a clinical model to explore factors associated with PSC development and recurrence [89–91]. IBD appears to adversely affect post-transplant outcomes. The risk of graft failure in patients with active IBD at the time of LT has been reported to be approximately tenfold higher than in those with inactive IBD [92]. In a 2019 meta-analysis of 14 studies, IBD was identified as an independent risk factor associated with a 1.7-fold higher risk of rPSC after LT [93]. When the timing of colectomy was considered in the same meta-analysis, colectomy performed before LT was associated with a reduced risk of rPSC, with a hazard ratio of 0.65 [93]. However, a systematic review [94] noted that although some studies identified an intact colon at transplantation as a risk factor for rPSC [95, 96], results were not consistent across studies, indicating limited evidence to support routine colectomy solely for the prevention of rPSC. Moreover, persistence or de novo development of UC after LT has been reported as an independent risk factor for rPSC, with a hazard ratio of 2.40 [90], underscoring the importance of appropriate control of post-transplant colitis activity.

The direction and magnitude of changes in IBD activity after LT also remain variable. While some studies have reported decreased IBD activity and fewer relapses after LT [80, 97], others have suggested that 27–59% of patients experience worsening disease activity [98, 99]. Up to 18% of patients develop IBD within 10 years after LT [100], a rate comparable to the incidence of clinically apparent IBD after PSC diagnosis [1, 2]. Multiple factors are likely to influence the post-transplant IBD course, including differences in immunosuppressive regimens, biliary anastomotic complications, infections, and concomitant IBD therapies, but studies that disentangle the relative contributions of these factors remain limited.

### Colorectal neoplasia

In PSC, which is frequently accompanied by colitis, the risk of colorectal cancer (CRC) has been reported to be increased by approximately sevenfold compared with the general population [101], and multiple cohorts have shown that PSC-IBD carries a higher risk of colorectal neoplasia than UC alone [13, 19, 20]. Recent data further suggest that even PSC without concomitant IBD may confer an increased CRC risk compared with the general population [102]. CRC in PSC-IBD tends to arise more frequently in the proximal colon, where inflammation is often most prominent [13, 100], consistent with the characteristic right-sided inflammatory distribution observed in this condition [80].

Dysplasia arising in PSC-IBD is often multifocal and may present as flat or otherwise atypical lesions. An increased prevalence of non-conventional dysplasia and invisible dysplasia has been reported, and such lesions can be difficult to detect using standard white-light endoscopy alone [103]. Moreover, even in clinical remission, endoscopic and histologic inflammation often persists in the right colon in PSC-IBD [26]. This mild background inflammation may reduce lesion conspicuity and complicate histologic assessment, thereby making early visible dysplasia detection more challenging [104, 105]. Collectively, these features likely contribute to the increased risk of advanced neoplasia at diagnosis and provide a rationale for more intensive surveillance strategies than those employed in conventional UC.

Accordingly, major international guidelines for PSC recommend ileocolonoscopy with segmental biopsies at the time of PSC diagnosis, even in the absence of known IBD, to evaluate for asymptomatic colitis (Table 2). In patients with PSC in whom IBD is not identified on the initial examination, repeated colonoscopy at approximately 5-year intervals is recommended or considered, in the absence of symptoms, to screen for incident IBD and colorectal neoplasia [11, 16, 106]. For patients with extensive UC or CD without PSC, most guidelines recommend initial screening colonoscopy approximately 8 years after diagnosis, followed by surveillance at 1–5-year intervals depending on individual risk [104, 107, 108]. By contrast, PSC-IBD is consistently positioned as a high-risk group for CRC in both IBD and hepatology guidelines and is treated as an exception to the conventional 8-year rule. Surveillance is therefore recommended to begin at the time of PSC-IBD diagnosis, with colonoscopy performed approximately annually [11, 16, 104, 106, 107, 109, 110]. With respect to endoscopic technique, current guidelines and consensus statements recommend high-definition colonoscopy as the standard approach, supplemented by dye-based chromoendoscopy or virtual chromoendoscopy for enhanced inspection, with targeted biopsies of visible lesions rather than random sampling [11, 111, 112].

**Table 2:** International Guideline Recommendations for Colonoscopic Surveillance in PSC

	Guideline	At PSC diagnosis	PSC without IBD	PSC-IBD
Europe	EASL 2022 [16]	Ileocolonoscopy with biopsies from all colonic segments including the terminal ileum, regardless of the presence of lesions	Colonoscopy every 5 years or whenever symptoms suspicious for IBD occur	Annual surveillance colonoscopy with biopsies (or every 1–2 years in selected patients without inflammatory activity)
	ECCO 2023 [108]	Not specifically addressed	Not specifically addressed	An annual surveillance colonoscopy should be performed following the diagnosis of PSC, irrespective of disease activity, extent, and duration
United States	AASLD 2023 [11]	Ileocolonoscopy with biopsies	Ileocolonoscopy every 5 years or whenever symptoms suspicious for IBD occur	High-definition surveillance colonoscopy with biopsies should start at age 15 years and be repeated at 1–2-year intervals
	AGA 2021 [104]	Initial colonoscopy screening should be performed immediately at the time of diagnosis	Not specifically addressed	1-year follow-up should be performed
Japan	PSC criteria 2024 [10]	Colonoscopy at the time of PSC diagnosis with particular attention to the presence of concomitant IBD	Not specifically addressed	Not specifically addressed
	JSCCR 2026 [110]	Not specifically addressed	Not specifically addressed	Annual surveillance colonoscopy beginning at diagnosis is recommended

Established factors influencing colorectal neoplasia risk in IBD include extent and activity of inflammation, disease duration, age at PSC diagnosis, and family history of CRC [113–115]. However, it remains unclear whether the right-sided predominant inflammatory distribution characteristic of PSC-IBD constitutes an independent risk factor for neoplasia. Studies directly evaluating the relationship between PSC-IBD endoscopic phenotype and colorectal neoplasia risk are limited, and phenotype-based risk stratification therefore remains an important priority for future research.

### Cholangiocarcinoma

Patients with PSC are at high risk for hepatobiliary malignancies, most notably cholangiocarcinoma, with a reported lifetime risk of biliary tract cancer of 7–15%, primarily cholangiocarcinoma [12, 13]. Cholangiocarcinoma accounts for nearly one third of PSC-related deaths [116], and approximately one third of cases are diagnosed within the first year after PSC diagnosis [12]. Because many tumors are unresectable at presentation, early detection and effective risk stratification remain major clinical priorities that critically influence long-term outcomes. Accordingly, surveillance for hepatobiliary malignancies represents an essential component of PSC management, alongside colorectal neoplasia surveillance. For hepatobiliary malignancy surveillance, periodic imaging using abdominal ultrasonography and contrast-enhanced magnetic resonance imaging with MRCP, together with measurement of serum CA19-9, is commonly performed in clinical practice. When clinically indicated, biliary surveillance using ERCP, including brush cytology, has also been evaluated [11, 16].

However, whether concomitant IBD itself constitutes an independent risk factor for cholangiocarcinoma in PSC remains uncertain, as findings across studies have been inconsistent. Several reports suggest that other factors, including older age, the presence of dominant biliary strictures, and disease duration of IBD, are more consistently associated with cholangiocarcinoma risk than IBD status itself [117–119]. In addition, dysbiosis, altered BA metabolism, and disruption of gut–liver axis immune regulation in PSC have been hypothesized to contribute to cholangiocarcinogenesis [120–122]; however, current evidence is largely hypothesis-generating and derived primarily from observational and preclinical studies.

### Pharmacological therapy

Although LT remains the only curative option for PSC, current management is largely supportive and aims to alleviate cholestasis-related symptoms and complications, including endoscopic interventions when indicated. Ursodeoxycholic acid (UDCA) is commonly prescribed to improve cholestasis; however, although standard doses of 13–15 mg/kg/day improve serum biochemical markers, benefit on clinical outcomes, including transplant-free survival, has not consistently been demonstrated [123]. Moreover, high-dose UDCA at 28–30 mg/kg/day has been associated with serious adverse events and worse outcomes [124]. Immunosuppressive agents, including corticosteroids, thiopurines, and cyclosporine [125], as well as TNF inhibitor [126], have shown no efficacy in PSC. With respect to intestinal disease activity, a small prospective single-center study suggested that UDCA may exert favorable effects on intestinal inflammation [127], and similar benefits have been reported in murine models [128–130].

In recent years, clinical development has focused on agents targeting BA signaling and transport. Therapies of particular interest that have advanced to phase III include pan-peroxisome proliferator-activated receptor agonist (bezafibrate), being evaluated as add-on therapy to UDCA (BEZASCLER; NCT04309773) [131, 132], and norursodeoxycholic acid (norUDCA), which has progressed from earlier phase II biochemical signals to a pivotal phase III program (NUC-5; NCT03872921) [133]. Overall, evidence that these therapies improve hard endpoints, such as transplant-free survival, remains limited, and none has reached the level of recommendation as standard therapy at present [134, 135].

As antimicrobial approaches, prospective clinical trials have evaluated oral antibiotics, such as metronidazole and vancomycin, and have demonstrated significant reductions in serum alkaline phosphatase [67, 136, 137]. In contrast, concerns remain regarding adverse consequences of long-term antibiotic use, including exacerbation of dysbiosis and emergence of antimicrobial resistance. In this context, bacteriophage therapy, which selectively targets specific pathogenic bacteria, has attracted renewed attention. Owing to their specificity, bacteriophages are expected to exert minimal effects on commensal microbiota, potentially limiting microbiome perturbation and resistance development. In murine models of liver disease, bacteriophages targeting *Enterococcus faecalis* implicated in alcohol-associated hepatitis improved hepatic inflammation [138], while phages targeting alcohol-producing *Klebsiella pneumoniae* implicated in metabolic dysfunction-associated steatotic liver disease similarly ameliorated liver injury [139]. Our group is developing bacteriophages targeting *Klebsiella pneumoniae* isolated from patients with PSC and demonstrated the efficacy of a phage cocktail and its therapeutic potential in a murine model of biliary injury [78]. Continued advances in microbiome research, together with mechanistic dissection of gut–liver axis pathways, are expected to facilitate clinical translation in diagnostics and therapeutics.

For PSC-IBD, treatment algorithms generally follow established guidelines for UC and CD, and standard IBD therapies are used, including 5-ASA, corticosteroids, thiopurines, biologic agents, and small-molecule inhibitors. However, therapeutic decision-making requires consideration of PSC-specific factors beyond conventional IBD algorithms (Table 3). In this cholestatic liver disease, drug-induced liver injury may be difficult to distinguish from PSC progression, necessitating careful monitoring of liver biochemistry, particularly given that many therapeutic agents undergo hepatic metabolism. PSC is also associated with recurrent bacterial cholangitis, and cumulative immunosuppression, particularly with combination therapy [142], may increase the risk of infectious complications. Given the progressive nature of PSC, potential liver transplantation should be incorporated into long-term treatment planning. Before LT, cumulative immunosuppression and malignancy risk may influence transplant eligibility. After LT, careful coordination with the transplant team is required to avoid excessive overlap between IBD-directed therapy and post-transplant immunosuppression. Notably, tacrolimus has demonstrated efficacy in UC [141], and may therefore have therapeutic relevance for UC management in this setting. In patients with advanced fibrosis or cirrhosis, altered drug metabolism and increased susceptibility to adverse events warrant individualized dosing and close monitoring. Furthermore, because PSC confers an elevated baseline risk of colorectal and hepatobiliary malignancies, systemic immunosuppression should be individualized, particularly in patients with a history of or high risk for malignancy. Despite these considerations, no consensus has been reached regarding the impact of colitis-directed therapy on the course of PSC. In a nationwide Swedish cohort, UDCA exposure was not associated with reduced mortality, whereas statin and thiopurine exposure were associated with improved survival and/or transplant-free outcomes [142]. In contrast, a French nationwide cohort found no significant association between thiopurine exposure and reduced risk of LT [143], highlighting heterogeneity across cohorts and endpoints. In parallel with major advances in IBD therapeutics, increasing attention has been directed toward the potential hepatic effects of newer biologics and small-molecule agents. Current UC therapies include TNF inhibitors (infliximab, adalimumab, golimumab), integrin inhibitors (vedolizumab, carotegrast methyl), interleukin-12/23 inhibitors (ustekinumab), interleukin-23 inhibitors (mirikizumab, risankizumab, guselkumab), Janus kinase inhibitors (tofacitinib, upadacitinib, filgotinib), and sphingosine-1-phosphate receptor modulators (ozanimod, etrasimod). The effects of these agents on PSC activity have primarily been examined in retrospective cohorts of patients with IBD and concomitant PSC. In a small randomized trial, infliximab showed no effect on endpoints including alkaline phosphatase [126]. Several retrospective studies likewise failed to identify a protective effect of infliximab, adalimumab, or vedolizumab on concomitant PSC [144–146]. Tofacitinib has been associated with lower serum alkaline phosphatase levels in retrospective analysis [147]; however, long-term outcome data remain limited across studies [148], and further validation is warranted.

**Table 3:** PSC-Specific Considerations for Therapeutic Selection in PSC-IBD

Clinical consideration for PSC	Key implications in PSC-IBD
Hepatotoxicity	Monitor liver biochemistry and distinguish drug-induced liver injury from PSC progression in the setting of underlying cholestatic liver disease
Infection risk	Carefully weigh cumulative immunosuppression, particularly combination therapy, in the context of cholangitis risk
Liver transplantation (LT)	Incorporate potential liver transplantation into long-term management and consider cumulative immunosuppression and malignancy risk before LT while avoiding excessive overlap between IBD-directed and post-transplant immunosuppression after LT in coordination with the transplant team
Cirrhosis	Adjust dosing and monitor closely in patients with advanced liver disease
Malignancy risk	Individualize systemic immunosuppression, particularly in patients with a history of or high risk for malignancy

## Summary and future directions

In this review, drawing on prior reports and our own data, we outline that PSC-IBD exhibits a characteristic endoscopic phenotype distinct from conventional UC, including a right-sided predominant distribution of colonic inflammation and high frequencies of RS and BI. We further summarize evidence indicating that PSC-IBD differs from conventional UC at the level of disease substrates, encompassing genetic susceptibility, BA signaling, gut microbiota composition, and immune responses mediated through the gut–liver axis. Collectively, these observations support the concept that PSC-IBD should be viewed not merely as a subtype of conventional UC, but as a distinct colitis phenotype pathophysiologically linked to PSC.

From a long-term outcomes perspective, multiple cohorts have demonstrated an increased risk of CRC in PSC-IBD. Accordingly, comprehensive management is required, integrating full ileocolonoscopy at the time of PSC diagnosis to evaluate the presence and extent of colitis, intensified CRC surveillance at short intervals, appropriate control of intestinal inflammation, and longitudinal assessment of biliary disease. Future studies should aim to establish more refined and individualized management strategies by integrating biliary and intestinal disease activity and mechanistic insights into bile-acid-microbiota-immune interactions.

## Abbreviations

BA: Bile acid
BI: Backwash ileitis
CCL25: C–C motif chemokine ligand 25
CCR9: C–C motif chemokine receptor 9
CD: Crohn’s disease
CRC: Colorectal cancer
ERCP: Endoscopic retrograde cholangiopancreatography
FGF19: Fibroblast growth factor 19
FXR: Farnesoid X receptor
HLA: Human leukocyte antigen
IBD: Inflammatory bowel disease
LT: Liver transplantation
MAdCAM-1: Mucosal vascular addressin cell adhesion molecule 1
MRCP: Magnetic resonance cholangiography
norUDCA: Norursodeoxycholic acid
p-ANCA: Perinuclear anti-neutrophil cytoplasmic antibody
PSC: Primary sclerosing cholangitis
PSC-IBD: Primary sclerosing cholangitis-associated inflammatory bowel disease
rPSC: Recurrent primary sclerosing cholangitis
RS: Rectal sparing
TGR5: Transmembrane G protein-coupled receptor 5
TNF: Tumor necrosis factor
UC: Ulcerative colitis
UDCA: Ursodeoxycholic acid
5-ASA: 5-Aminosalicylic acid
